# Construction of Porous Carbon Nanosheet/Cu_2_S Composites with Enhanced Potassium Storage

**DOI:** 10.3390/nano13172415

**Published:** 2023-08-25

**Authors:** Meiqi Mu, Bin Li, Jing Yu, Jie Ding, Haishan He, Xiaokang Li, Jirong Mou, Jujun Yuan, Jun Liu

**Affiliations:** 1 College of Physics and Electronics, Gannan Normal University, Ganzhou 341000, China; mumeiqi77@163.com (M.M.); yjyj131313@163.com (J.Y.); dingjie321823@163.com (J.D.); lixiaokang@gnnu.edu.cn (X.L.); mjr08024@163.com (J.M.); 2 Ganzhou Jirui New Energy Technology Co., Ltd., Ganzhou 341000, China; wpslibin@163.com; 3 College of Chemistry and Chemical Engineering, Gannan Normal University, Ganzhou 341000, China; hhs9894@163.com; 4 School of Materials Science and Engineering, South China University of Technology, Guangzhou 510641, China

**Keywords:** potassium-ion battery, anode material, Cu_2_S, porous carbon nanosheet, electrochemical performance

## Abstract

Porous C nanosheet/Cu_2_S composites were prepared using a simple self-template method and vulcanization process. The Cu_2_S nanoparticles with an average diameter of 140 nm are uniformly distributed on porous carbon nanosheets. When used as the anode of a potassium-ion battery, porous C nanosheet/Cu_2_S composites exhibit good rate performance and cycle performance (363 mAh g^−1^ at 0.1 A g^−1^ after 100 cycles; 120 mAh g^−1^ at 5 A g^−1^ after 1000 cycles). The excellent electrochemical performance of porous C nanosheet/Cu_2_S composites can be ascribed to their unique structure, which can restrain the volume change of Cu_2_S during the charge/discharge processes, increase the contact area between the electrode and the electrolyte, and improve the electron/ionic conductivity of the electrode material.

## 1. Introduction

Lithium-ion batteries (LIBs) have been proven to be effective energy storage devices for portable electronic products, smart grids, and electric vehicles. Particularly as the social economy continues to grow rapidly, there is an increasing demand for LIBs [1]. However, lithium resources have low reserves in the Earth’s crust and an uneven distribution, which limits their future large-scale energy storage applications. Given the circumstances, it is imperative to develop a new battery technology with excellent performance, abundant resources, and a low price [2,3].

The plentiful presence of potassium in the Earth’s crust offers promising economic prospects for potassium-ion batteries (PIBs) [4,5]. In the periodic table of elements, potassium is in the same main group as lithium and they have similar chemical properties. For example, the standard hydrogen potential of potassium is −2.93 V vs. Eo, while that of lithium is −3.04 V vs. Eo; the two values are very similar, which means that PIBs also have relatively high voltage and energy density [6,7,8]. PIBs also possess a “rocking chair” energy storage behavior similar to LIBs. Therefore, PIBs can be used as a potential alternative to LIBs [9,10]. However, due to the large radius of potassium ions (0.138 nm), the frequent insertion/extraction of K^+^ within charge/discharge processes can lead to large volumetric expansion of the anode material, resulting in capacity loss, cycle instability, and poor rate performance [11,12,13]. Thus, developing a kind of effective anode material for PIBs is urgent.

Carbon materials are abundant, cheap, non-toxic, and environmentally friendly, and they have exhibited good cycle stability for potassium-ion battery anode materials. The potassium storage mechanisms of carbon materials are mainly divided into two types. One is the reversible insertion/extraction potassium ion mechanism for carbon materials. Jian et al. [14] studied the deintercalation mechanism of potassium ions in graphite by using in situ XRD technology and found that potassium ions undergo a third-order phase transition when embedded in graphite and undergo the KC_36_–KC_24_–KC_8_ transition process, where the theoretical specific capacity is 279 mAh g^−1^. Feng et al. [15] used expanded graphite as the anode of potassium-ion batteries, which maintained a reversible capacity of 174 mAh g^−1^ at a current density of 200 mA g^−1^. However, graphite, as the anode material of potassium-ion batteries, is prone to deformation of the material structure during the charging and discharging processes, and the volume changes greatly, resulting in rapid attenuation of battery capacity [16,17]. The other is potassium storage by insertion/extraction and adsorption/desorption mixing mechanisms, which refer to potassium-ion storage between carbon layers by insertion/extraction at low potential, and surface-induced capacitance to store potassium through adsorption/desorption on nanopores and surface defects/functional groups at high potential. Such potassium storage processes will occur in doped and porous carbon materials. Hard carbon materials are usually rich in porosity and structural disorder, and their synthesis is simple and controllable. The abundant pores and disordered structure facilitate the insertion and extraction of potassium ions with large radii [18]. At the same time, unlike graphite, hard carbon has a large layer spacing and can still maintain structural integrity during continuous potassium storage. For example, Liu et al. [19] prepared graded porous hard carbon from low-cost and abundant soybeans. Thanks to the medium surface area, low graphitization and large layer spacing of the graded porous hard carbon, it exhibits a reversible capacity of 70 mAh g^−1^ at a current density of 800 mA g^−1^. Tian et al. [20] synthesized sulfur-doped bamboo carbon anode materials that can meet the intercalation and extraction of potassium ions using biomass bamboo charcoal as a precursor and sulfur as a modified element. The material is activated, carbonized and vulcanized at 700 °C to form a unique structure that exhibits stable electrochemical properties. The reversible capacity at a current density of 1 A g^−1^ is 124.2 mAh g^−1^. The results show that the doping of heteroatoms can make carbon materials have more active sites, which can effectively improve the conductivity of ions and accelerate the transfer rate of ions, thereby improving the potassium storage performance of carbon materials, which helps electrode materials exhibit long cycle stability and good rate performance [21]. Nevertheless, the low cycling capacity of the carbon materials leads researchers to seek new anode materials.

As anode materials in the PIBs, transition metal sulfides (TMSs) such as FeS_2_, CoS, NiS_2_, and ZnS exhibit high theoretical capacity and redox reversibility [22,23,24,25]. Copper-based sulfides (Cu_x_S_y_) have garnered significant interest among transition metal sulfides because of their cost-effectiveness and higher conductivity (about 10^4^ S cm^−1^). However, copper-based sulfides still face issues such as greater volume expansion and slow kinetics of K^+^ in the cycling process, which lead to rapid capacity decay of copper-based sulfides and the fragmentation of electrode materials [26,27]. In order to overcome these issues, an effective strategy is proposed to construct copper-based sulfide/carbon nanocomposites (i.e., Cu_x_S_y_/C), which can suppress the volume change of Cu_x_S_y_, improve the diffusion kinetics of K^+^, and increase electronic conductivity, achieving the goal of improving cycle stability and rate performance [28,29,30,31,32]. For example, Huang et al. [28] synthesized three-dimensional ordered macroporous Cu_9_S_5_@C using metal–organic frameworks as sacrificial templates. The Cu_9_S_5_@C electrode material demonstrated a cycling capability of 316 mAh g^−1^ at 0.1 A g^−1^ after 200 cycles. Jia et al. [29] prepared CuS nanosheet@RGO composites, which displayed 311 mAh g^−1^ at 0.5 A g^−1^ after 100 cycles. Zhang et al. [30] fabricated a hollow nanocage heterostructure Cu_9_S_5_/MoS_2_/C composite, which displayed 350 mAh g^−1^ at 0.1 A g^−1^. Cao et al. [31] constructed a core–shell CuS-C@Nb_2_O_5_-C nanofiber (NFs) composite, which demonstrated 278.5 mAh g^−1^ at 0.1 A g^−1^. Although the described copper-based sulfide/C composites have shown improved electrochemical performance, their preparation involves complex experimental steps and requires a more stringent experimental equipment environment. These factors limit the widespread application and preparation of copper-based sulfide/C composite materials [33,34,35,36]. Therefore, it is extremely desirable to prepare a copper-based sulfide/C composite using a simple method for enhancing its electrochemical performance in PIBs.

In this work, the C/Cu_2_S composites are fabricated using a simple self-template method and vulcanization treatment. The introduction of carbon materials enhances the structural stability of Cu_2_S and improves the electronic conductivity of the composite. Experimental results show that the C/Cu_2_S composites exhibit excellent cycle performance and rate performance as the electrode material of PIB (363 mAh g^−1^ at 0.1 A g^−1^ upon 100 cycles; 120 mAh g^−1^ at 5 A g^−1^ upon 1000 cycles).

## 2. Experiment

### 2.1. Material Preparation

**Scheme 1:**
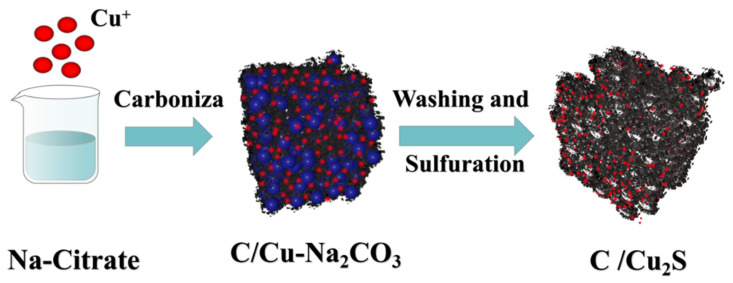
Schematic illustration of synthesis strategy for porous C nanosheet/Cu_2_S composites.

The synthesis process of porous C nanosheet/Cu_2_S composites is illustrated in Scheme 1. First of all, to obtain a clear and transparent solution, 0.5 mM (120.8 mg) of Cu(NO_3_)_2_·3H_2_O (98%, Aladdin) and 8 g of C_6_H_5_Na_3_O_7_ (≥98%, Aladdin) were dissolved in 40 mL of deionized water. The solution was subsequently dried to yield a precursor, and the precursor was further annealed at 700 °C for 2 h at a heating rate of 5 °C/min in an argon atmosphere. After the above annealing process, the intermediate C/Cu-Na_2_CO_3_ was acquired. To obtain the C/Cu composite, the intermediate was thoroughly washed with deionized water and ethanol to remove Na_2_CO_3_. Finally, to acquire porous C nanosheet/Cu_2_S composites, the C/Cu composites and S powder (weight ratio of 1:4) were poured into a mortar, evenly mixed, and annealed at 600 °C for 3 h at a heating rate of 5 °C/min in an argon atmosphere.

### 2.2. Material Characterization

The crystalline structure of porous C nanosheet/Cu_2_S composites was characterized using powder X-ray diffraction (XRD, DX2700, Dandong Haoyuan Instrument Co., Ltd., Dandong, China) at a current density of 45 mA and an operation voltage of 40 kV with Cu Kα radiation and Raman spectra (Lab RAMHR800, HORIBA Jobin Yvon Company, Longjumeau, France). The weight ratio of Cu_2_S within C/Cu_2_S composites was examined through the TGA (TA–Q600, TA Instruments Company, New Castle, DE, America) system conducted in an oxygen atmosphere at a heating rate of 10 °C min^−1^ from 30 °C to 700 °C. The morphology and microstructure of porous C nanosheet/Cu_2_S composites were investigated using field emission scanning electron microscopy (SEM, FEI Quanta 200, FEI Company, Hillsboro, OH, America) and transmission electron microscopy (TEM, JEM–2100F, JEOL Company, Mitaka, Japan). Additionally, the chemical composition of the porous C nanosheet/Cu_2_S composites was determined through X-ray photoelectron spectroscopy (XPS, K-Alpha 1063, Thermo Scientific Company, Waltham, MA, America) with an Al Κα X-ray source. The Brunauer–Emmett–Teller (BET) specific surface area of porous C nanosheet/Cu_2_S composites was studied using a Quadrachrome Adsorption Instrument (BSD 660, BEST Instruments, Beijing, China).

### 2.3. Electrochemical Measurement

The CR2032 coin cell was used to assess the electrochemical performance of C/Cu_2_S composites. For fabricating the working electrode, the C/Cu_2_S composite, acetylene black, and sodium alginate (mass ratio: 7:2:1) were mixed with deionized water to form a black slurry. The mixed slurry was coated on the copper foil and dried at 80 ℃ for 12 h. The mass loading of the C/Cu_2_S working electrode is about 0.9–1.3 mg cm^−2^. The electrolyte was 1 M KFSl in EC:DEC (1:1 Vol%), and the counter electrode is made of a potassium plate. The assembling procedures for half cells took place in an Ar-filled glovebox. The NEWARE battery test system was used to test the galvanostatic charge/discharge properties of porous C nanosheet/Cu_2_S composites. Cyclic voltammetry (CV) and electrochemical impedance spectroscopy (EIS) tests were performed on the electrochemical workstation (VMP3). The voltage window of cyclic voltammetry is 0.01–3.0 V (relative to K/K^+^), the frequency range of electrochemical impedance spectroscopy is 100 kHz to 100 mHz, and the amplitude is 0.005 V.

## 3. Results and Discussion

The synthesis of porous C nanosheet/Cu_2_S composites is shown in Scheme 1. Typically, the intermediate C/Cu-Na_2_CO_3_ was first fabricated by simple mixing and an annealing process. During the annealing process, the organic ligands of sodium citrate molecules are converted into carbon dots at relatively low annealing temperatures in an inert atmosphere. Meanwhile, the Na^+^ species in sodium citrate molecules will be in situ turned into hexagonal Na_2_CO_3_ crystals. With increasing temperatures, these Na_2_CO_3_ crystals can act as catalysts and hard templates for the subsequent growth of network-like carbonization product at higher temperatures. When the temperature was increased to 700 °C, ultrathin carbon sheets were formed on the surfaces of Na_2_CO_3_ crystals, while Cu(NO_3_)_2_·3H_2_O was transformed into Cu nanoparticles and embedded on the surface of the carbon layers [37,38]. For obtaining the porous C nanosheet/Cu_2_S composites, the intermediate C/Cu-Na_2_CO_3_ was thoroughly washed with deionized water and ethanol to remove Na_2_CO_3_. Finally, the porous C nanosheet/Cu_2_S composites were fabricated using the solid sulfidation process of the porous C nanosheet/Cu_2_S template.

**Figure 1:**
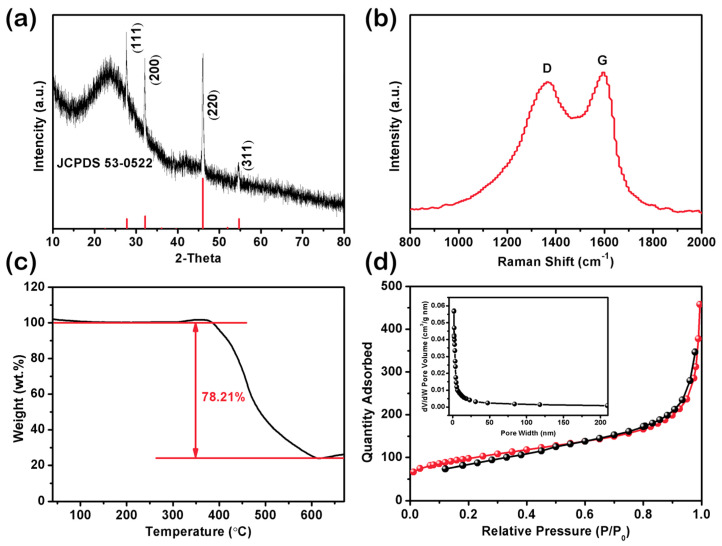
(**a**) XRD patterns of C/Cu_2_S. (**b**) Raman spectroscopy of C/Cu_2_S. (**c**) TG curves of C/Cu_2_S. (**d**) N_2_ adsorption–desorption isotherms and the corresponding pore size distribution of C/Cu_2_S.

**Figure 2:**
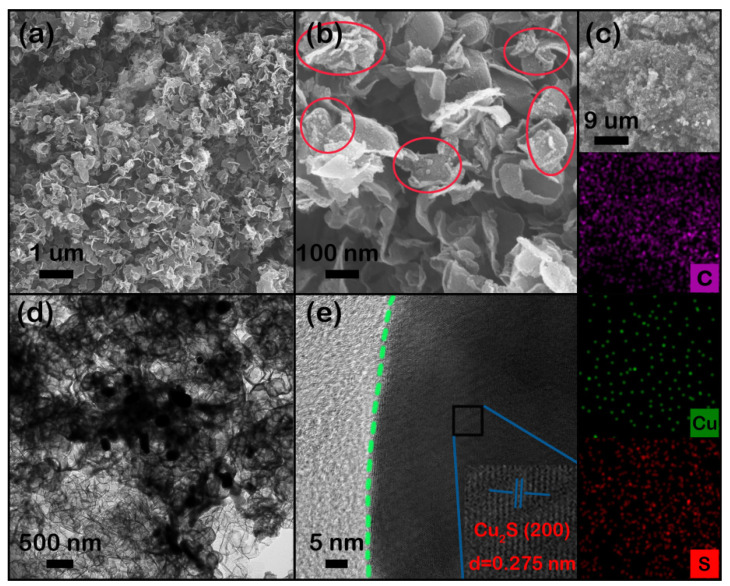
(**a**,**b**) SEM images of C/Cu_2_S. (**c**) Element mappings of C/Cu_2_S. (**d**,**e**) TEM and HR-TEM images of C/Cu_2_S.

The XRD pattern of the C/Cu_2_S composite shows that the diffraction peaks at 2θ = 27.71°, 32.14°, 46.09°, and 54.69° correspond to the (111), (200), (220), and (311) crystal planes of Cu_2_S (JCPDS 53-0522) (Figure 1a) [39]. The Raman spectra of the C/Cu_2_S composite shows two distinct peaks at 1366 cm^−1^ and 1594 cm^−1^, corresponding to the defective carbon and graphitized carbon, respectively (Figure 1b). A thermogravimetric analysis (TGA) was used to estimate the weight content of Cu_2_S. As shown in Figure 2c, a major weight change takes place between 320 and 380 °C. Therein, the weight increase before 380 °C is due to the formation of CuO during the oxidation of Cu_2_S. With a further increase in temperature, the significant weight decrease is due to the burning of the carbon layer. The weight loss rate of C/Cu_2_S is 78.21%. According to the reaction equation (Cu_2_S + 2O_2_ = 2CuO + SO_2_↑; C + O_2_ = CO_2_↑), the weight content of Cu_2_S in C/Cu_2_S is calculated to be 12%. As observed in Figure 1d, the Brunauer–Emmett–Teller (BET) specific surface area of C/Cu_2_S is 346.47 m^2^ g^−1^, and the average pore size is 7.72 nm. The presence of a porous structure not only allows for accommodating volume changes during cycling but also facilitates ion conduction.

The morphology of the product was observed within SEM images (Figure 2a,b). The C/Cu_2_S composite presents a sheet structure with a thickness of 10–20 nm. The Cu_2_S nanoparticles can be seen in the red circled part (Figure 2b). The elemental mapping images show the elements (C, Cu, and S) were evenly distributed in C/Cu_2_S composites (Figure 2c). As displayed within the TEM image (Figure 2d), it can be seen that Cu_2_S nanoparticles with a diameter of 140–180 nm are dispersed on C nanosheets. From the HRTEM image analysis, it is observed that the interplanar spacing is measured at 0.275 nm, which corresponds to the (200) plane of Cu_2_S (Figure 2e).

**Figure 3:**
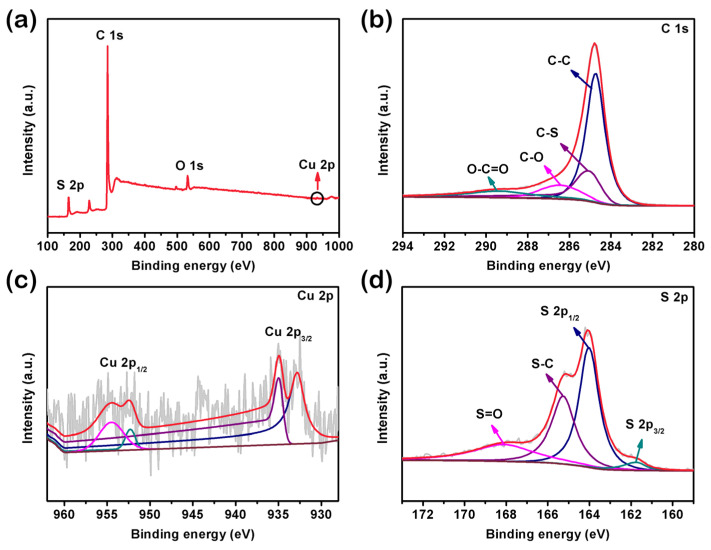
High–resolution XPS spectra of (**a**) survey spectra, (**b**) C 1s, (**c**) Cu 2p, and (**d**) S 2p elements for C/Cu_2_S.

XPS was further used to analyze the elements’ valence states in the C/Cu_2_S composites. Four peaks are shown in the survey spectrum, corresponding to the C, Cu, S, and O elements (Figure 3a). Among them, there is a weak Cu 2p signal peak at 935 eV, which is ascribed to the low content of Cu in the C/Cu_2_S composite. For the C 1s spectrum (Figure 3b), the peaks at 284.78, 285.11, 286.51, and 289.57 eV belong to C–C/C=C, C–S, C-O, and O–C=O, respectively [40]. For the Cu 2p spectrum (Figure 3c), the two peaks centered at 932.27 and 951.8 eV belong to Cu 2p_3/2_ and Cu 2p_1/2_ Cu^+^, while the peaks at 934.8 and 954.3 eV can correspond to the Cu 2p_3/2_ and Cu 2p_1/2_ of Cu^2+^, respectively [41]. For the S 2p spectrum (Figure 3d), the two peaks at around 161.77 and 164.03 eV are assigned to S 2p_3/2_ and S 2p_1/2_, respectively, and the other two peaks at 165.28 and 168.15 eV can correspond to S–C and SO_x_, respectively [42,43].

**Figure 4:**
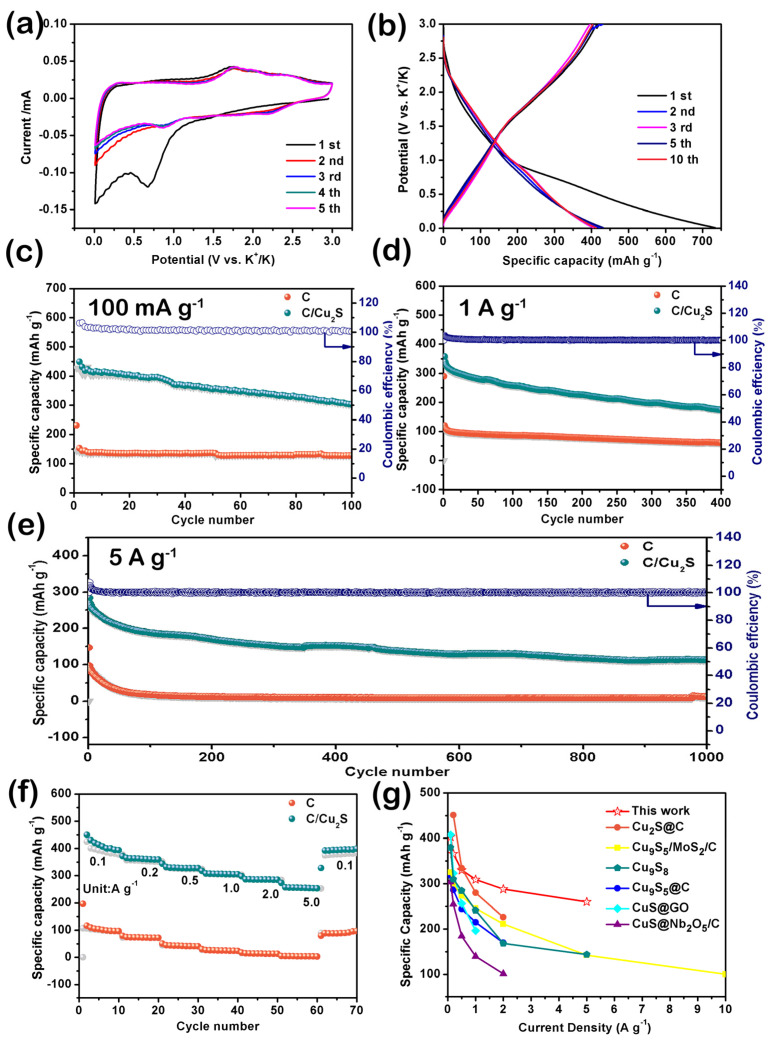
(**a**) CV curves for the first five cycles at a scan rate of 0.1 mV s^−1^. (**b**) Galvanostatic charge/discharge curves at 0.1 A g^−1^ of C/Cu_2_S. (**c**,**d**) Cycling stability of C/Cu_2_S and porous C at 0.1 A g^−1^ and 1 A g^−1^. (**e**) Long–term cycling stability at 5 A g^−1^. (**f**) Rate capability at different rates. (**g**) The performance of C/Cu_2_S compared with recently reported copper–based sulfide anode materials.

The C/Cu_2_S composites were tested in half cells to evaluate their electrochemical performance. The cyclic voltametric (CV) curves for the C/Cu_2_S composites were tested at a sweep speed of 0.1 mV s^−1^ over a voltage range of 0.01–3 V (Figure 4a). In the first cycle, there is a distinct broad peak between 0.4 and 0.7 V, which could be mainly attributed to the formation of a solid electrolyte interface (SEI) film and the conversion reaction of Cu_2_S to Cu metal and K_2_S_4_. An oxidation peak at 1.76 V could be related to the extraction of potassium ions and the reconstruction process of Cu_2_S [4,32,41]. After the first scan, the reduction/oxidation peaks of the CV curves in subsequent cycles remain highly similar, indicating that this is a reversible and stable reaction. Furthermore, the galvanostatic charge–discharge curves of the C/Cu_2_S composites were measured between 0.01 and 3 V at 0.1 A g^−1^. As observed in Figure 4b, the first discharge and charge capacities of C/Cu_2_S composites are 731 and 410 mAh g^−1^, respectively. The initial Coulomb efficiency was calculated at 56%. The initial capacity loss can be attributed to the formation of the SEI film and the occurrence of irreversible reactions [44]. It is evident to find that the galvanostatic charge–discharge curves of the following 2nd, 3rd, 5th and 10th are overlapped, which reflects the high stability of the C/Cu_2_S anode in PIBs. Figure S1 shows the charge–discharge curve of porous carbon at 0.1 A g^−1^. It can be seen the first discharge and charge capacities of C are 306 and 143 mAh g^−1^, respectively, which are significantly lower than that of C/Cu_2_S composites.

The cycling behaviors for C/Cu_2_S composites are displayed in Figure 4c–e. The C/Cu_2_S anode can maintain a reversible capacity of 363 mAh g^−1^ after 100 cycles at 100 mA g^−1^ (Figure 4c). In contrast, the capacity of the porous C electrode material is only 120 mAh g^−1^. Moreover, the C/Cu_2_S anode exhibits a reversible capacity of 221 mAh g^−1^ after 400 cycles at 1 A g^−1^, while the porous C electrode is 80 mAh g^−1^ (Figure 4d). When cycled at a high rate of 5 A g^−1^ for 1000 cycles, the C/Cu_2_S anode still renders a reversible capacity of 120 mAh g^−1^ (Figure 4e).

To further evaluate the cycling stability of the products, the rate performances of C/Cu_2_S composites and porous C were analyzed (Figure 4f). When the current density gradually increases from 0.1 to 5 A g^−1^, the reversible capacities of C/Cu_2_S are 403, 366, 330, 309, 288, and 260 mAh g^−1^, respectively. Subsequently, when the current density is reduced back to 0.1 A g^−1^, the reversible capacity of C/Cu_2_S recovers to 398 mAh g^−1^, indicating the excellent structural stability of the C/Cu_2_S composites. Notably, the remarkable rate capability of C/Cu_2_S is comparable to that of reported copper-based sulfides (Figure 4g and Table S1). It can be clearly seen that porous C nanosheet/Cu_2_S composites show the best rate property and cycling performance when compared with some other copper-based sulfide/carbon composites [28,29,32,36], Cu_9_S_5_/MoS_2_/C [30], and CuS@Nb_2_O_5_/C [31].

**Figure 5:**
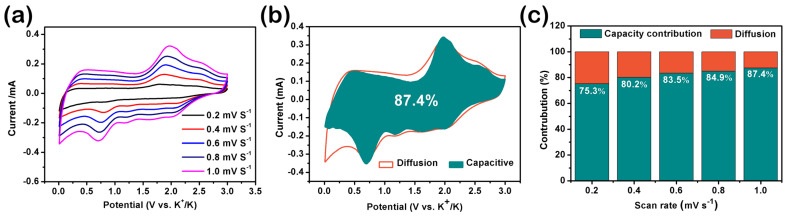
(**a**) CV curves of C/Cu_2_S with various scan rates of 0.2–1.0 mV s^−1^. (**b**) Pseudocapacitive contribution of C/Cu_2_S at a scan rate of 1.0 mV s^−1^. (**c**) Pseudocapacitive contribution ratios at different scan rates.

To determine the kinetic properties of C/Cu_2_S composites, the CV curves at different scan rates (0.2–1.0 mV s^−1^) are shown in Figure 5a. With the increase in scanning rate, these CV curves exhibit a similar trend. The contribution ratio of diffusion behavior and pseudocapacitive behavior can be demonstrated by Equation (1):

(1) $$i=k_{1}v+k_{2}v^{1/2}$$

The parameter *k*_1_*v* reflects the pseudocapacitive behavior, while the parameter *k*_2_*v*^1/2^ reflects the diffusion contribution. When integrating the CV curve (Figure 5b,c), the C/Cu_2_S composites achieved percentages of pseudocapacitive contributions of 75.3, 80.2, 83.5, 84.9, and 87.4% at 0.2, 0.4, 0.6, 0.8, and 1.0 mV s^−1^, respectively. Kinetic analysis shows that the pseudocapacitive contribution dominates the electrochemical process.

**Figure 6:**
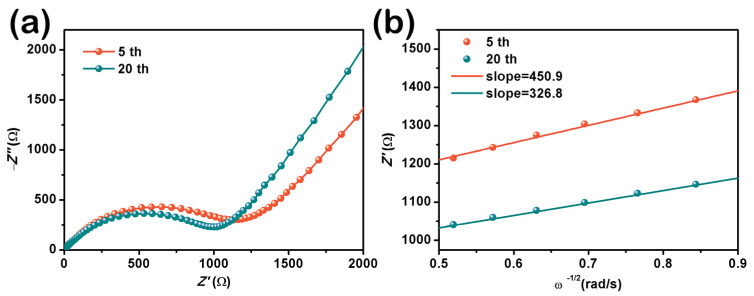
(**a**) EIS of C/Cu_2_S after 5 and 20 cycles. (**b**) Relationship profile between Zre and w^−1/2^ within low-frequency range for C/Cu_2_S.

EIS was employed to further investigate the reaction kinetics and k^+^ diffusion coefficient of C/Cu_2_s composites. In the Nyquist plot (Figure 6a), the sloped line and the semicircle represent the diffusion resistance of K^+^ and charge transfer impedance, respectively. Equations (2) and (3) were used to calculate the diffusion coefficient of K^+^:

(2) $$Z^{\prime }=R+\sigma_{\omega }\omega^{-1/2}$$

(3) $$D_{k}^{+}=R^{2}T^{2}/2n^{4}F^{4}\sigma_{\omega }^{2}A^{2}C^{2}$$

A *Z*′ and *ω*^−1/2^ relation graph is linearly fitted to obtain the slope *σ_w_*. After five cycles, the *σ_w_* value is calculated to be 450.9, and the corresponding K^+^ diffusion coefficient is 1.39 × 10^−13^ cm^2^ S^−1^. In Equations (2) and (3), *R*, *T*, *n*, *F*, *A*, *C*, and *σ_w_* are the gas constant (8.314 J mol^−1^ K^−1^), the absolute temperature (301.15 K), the number of electrons per molecule during oxidation, Faraday’s constant (96,485 C mol^−1^), the surface area of the electrode (1.13 cm^2^), and the K^+^ concentration in the electrode material (1.0 mol L^−1^), respectively. The *σ_w_* value after 20 cycles is 326.8, and the corresponding K-ion diffusion coefficient is 2.65 × 10^−13^ cm^2^ S^−1^. The larger the diffusion coefficient $D_{k}^{+}$, the faster the diffusion kinetics of potassium ions. This shows that its unique C/Cu_2_S composites can provide an efficient transport path for K ions.

## 4. Conclusions

In summary, the porous C nanosheet/Cu_2_S composites were successfully fabricated through a simple self-template method followed by a vulcanization process. When used as the anode material for PIBs, porous C nanosheet/Cu_2_S composites exhibit superior rate performance and cycling performance (363 mAh g^−1^ at 0.1 A g^−1^ after 100 cycles; 120 mAh g^−1^ at 5 A g^−1^ after 1000 cycles). The excellent electrochemical performance of porous C nanosheet/Cu_2_S composites can be ascribed to the unique structure, which can restrain the volume change of Cu_2_S during the charging and discharging processes, increase the contact area between the electrode and the electrolyte, and improve the electron/ionic conductivity of the electrode material. These results show that porous C nanosheet/Cu_2_S composites have good application prospects as anode materials for PIBs.

## Data Availability

Data will be made available on request.

## Supplementary Materials

The following supporting information can be downloaded at: https://www.mdpi.com/article/10.3390/nano13172415/s1. Table S1: Summary and comparison of cycling and rate performances of recently reported copper-based sulfide anode materials for PIBs; Figure S1: The galvanostatic charge–discharge profile of C at 0.1 A g^−1^.
