# Supplementary material for: Construction of Porous Carbon Nanosheet/Cu2S Composites with Enhanced Potassium Storage
Source: Nanomaterials (Basel). 2023 Aug 25;13(17):2415. doi: 10.3390/nano13172415 (PMC10489898; doi:10.3390/nano13172415)
Supplement: Supplementary file 1 [file nanomaterials-13-02415-s001.zip › nanomaterials-2510974-supplementary.pdf]

Supporting Information

Table S1. Summary and comparison of cycling and rate performances of recently reported copper-based sulfides anode materials for PIBs.

Materials	Capacity retention (mAh g^{-1})	Rate Performance Capacity (mAh g^{-1})	Rate Performance Current (mA g^{-1})	Reference
C/Cu_2S	363 (100 cycles) at 0.1 A g^{-1}; 120 (1000 cycles) at 5 A g^{-1}	403, 366, 330, 309, 288, 260	0.1, 0.2, 0.5, 1, 2, and 5	This work
Cu_9S_5@C	316 (200 cycles) at 0.1 A g^{-1}	318, 286, 244, 215, and 170	0.1, 0.2, 0.5, 1, and 2	[28]
CuS@GO	311 (100 cycles) at 0.5 A g^{-1}	407.7, 322, 256.2, and 196.5	0.1, 0.2, 0.5, and 1	[29]
Cu_9S_5/MoS_2/C	270.6 (200 cycles) at 0.5 A g^{-1}	325, 300.2, 273.5, 246, 211, 142.3, and 100.2	0.1, 0.2, 0.5, 1, 2 ,5, and 10	[30]
CuS@Nb_2O_5/C	278.5 (100 cycles) at 0.1 A g^{-1}	304, 254.5, 184.4,139.6, and 101.4	0.1, 0.2, 0.5, 1, and 2	[31]
Cu_2S@C	206.6 (400 cycles) at 2 A g^{-1}	451.2, 334, 280.1, 226.3	0.2, 0.5, 1, and 2	[32]
CNT/Cu_9S_8	320.5 (200 cycles) at 0.1 A g^{-1}	380, 309.4, 284.5, 241, 168.5, 144	0.1, 0.2, 0.5, 1, 2, and 5	[36]

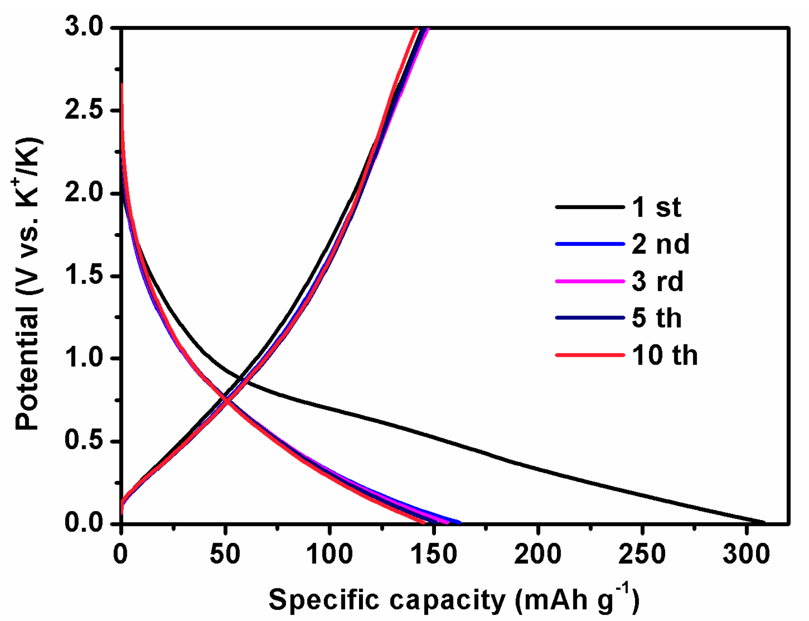

Figure S1: The galvanostatic charge-discharge profile for porous carbon at 0.1 A g^{-1}.
